# IL‐17A Mediates Depressive‐Like Symptoms by Inducing Microglia Activation in Psoriasiform Dermatitis Mice

**DOI:** 10.1002/iid3.70092

**Published:** 2024-12-11

**Authors:** Yue Dou, Jingjing You, Jing Wang, Xinxin Li, Yawen Lin, Bin Liu, Lei Ma

**Affiliations:** ^1^ Department of Dermatology Binzhou Medical University Hospital Binzhou Shandong China; ^2^ Department of Medical Research Center Binzhou Medical University Hospital Binzhou Shandong China

**Keywords:** depression, IL‐17A, microglia, psoriasis

## Abstract

**Background:**

Psoriasis is recognized as a systemic disease for its accompanying comorbidities, among which psychological disorders present a high incidence rate and affect patients’ life quality. Interleukin (IL)‐17A is the central pathological factor in the pathogenesis and development of psoriasis.

**Objective:**

To clarify if psoriasis‐induced systemic IL‐17A increase can mediate the neuronal inflammation and result in depressive‐like symptoms.

**Methods:**

Psoriasiform dermatitis model was established by imiquimod (IMQ) application on male BALB/c mice and IL‐17A intervention was performed by lateral ventricular catheterization. Skin structural, histopathological characteristics, and behavioral tests were assessed. Serum IL‐17A levels were detected by Enzyme‐linked immunosorbent assay. mRNA expression of pro‐inflammatory factors IL‐1β, IL‐6, and tumor necrosis factor‐α (TNF‐α) as well as anti‐inflammatory factors IL‐4 and IL‐10 in the hippocampus and cortex were measured by RT‐qPCR. The number of microglia and hippocampal neurons was quantified by immunofluorescent assay.

**Results:**

IMQ treatment resulted in significant skin structural and histopathological characters of psoriasiform dermatitis with elevated serum IL‐17A levels, obvious depressive‐like behaviors, microglia activation with increased IL‐1β, IL‐6, and TNF‐α expression levels in the hippocampus and cortex, and notable inhibition of hippocampal neurogenesis. While, IL‐17A neutralization by intracerebroventricular injection of anti‐IL‐17A antibody can remarkably inhibit microglia activation and decrease the abnormally increased expression levels of IL‐1β, IL‐6, and TNF‐α in the hippocampus and cortex of psoriasiform dermatitis mice, promote hippocampal neurogenesis, thus alleviate the depressive‐like behaviors.

**Conclusion:**

In the pathological condition of psoriasis, systemic IL‐17A elevation can trigger microglia activation, provoke pro‐inflammation mediators to release, evoke neuroinflammation, subsequently inhibit hippocampal neurogenesis, and result in depression. IL‐17A, as an important pathogenic factor in psoriasis, contributes to its critical role in mediating systemic inflammation and depression comorbidity.

## Graphical abstract

In the pathological condition of psoriasis, systemic IL‐17A elevation can trigger microglia activation, provoke pro‐inflammation mediators release, evoke neural inflammation effects, then inhibit hippocampal neurogenesis and result in depression development.

## 1 Introduction

Psoriasis is an immune‐mediated chronic recurrent inflammatory skin disease that affects about 2%–3% of the worldwide adult population. The most common clinical manifestation is psoriasis vulgaris, accounting for approximately 80%–90% of cases and presenting as symmetrical, erythematous, scaling papules and plaques [1]. In recent years, psoriasis has been recognized as not only a skin disease, but also a systemic disease, since it also causes arthritis, kidney disease, gastrointestinal disease, cardiovascular disease, metabolic syndrome, psychological disorder, and so on [2]. Psoriasis can isolate its patients, in which the unacceptable appearance of psoriatic lesions can cause discomfort and may increase unemployment and layoff rates, as well as depressive states up to suicide rates [3, 4]. So, both physical and psychosocial impacts of psoriasis are considerable and negatively affect patients’ life quality [5]. Moreover, the prevalence of psychological disorders in psoriasis patients is even higher than in patients with other dermatological conditions, presenting an obviously increased risk of depression, anxiety, and suicidality [6, 7]. Though the comorbidity of psoriasis and depression does not appear to be merely coincidental, the mechanisms through which psoriatic clinical course may interfere with patients’ psychological comorbidity are unclear, improvements in the symptoms and signs are expected to alleviate patients’ psychological burden and improve their life quality. Emerging researches, both in mice and in humans, suggest that T helper 17 (Th17) cells and its important effective cytokine interleukin (IL)‐17A play a critical role in the development of psoriasis, which overexpress both in psoriatic lesions and peripheral circulation and associate with psoriasis disease severity [8, 9, 10]. And, targeting IL‐17A treatment has been demonstrated as an effective and guideline‐recommending therapy for moderate‐to‐severe plaque psoriasis [11, 12, 13, 14]. Recently, the effect of Th17 cells and IL‐17A in depression has been investigated, which is obviously augmented in the peripheral circulation of depression patients [15, 16]. Psoriasis patients with higher levels of Th17 cells and IL‐17A are more easily subjected to developing depression [6]. Encouragingly, the IL‐17A inhibitor treatment was demonstrated to be associated with a significant improvement in skin clearance and amelioration of depression symptoms in most patients with psoriasis [17]. Whether the amelioration of depression in psoriasis patients by IL‐17A inhibitor treatment is only a concomitant psychological change for their skin clearance or a positive result for specific IL‐17A targeting intervention? The possible mechanistic relationship between IL‐17A and its impact on depression symptoms in psoriasis patients has not been elucidated. During the psoriatic pathological process, whether the increased systemic IL‐17A levels can be responsible for its depression phenomenon by inducing neuronal inflammatory reaction is still a crucial issue to be explored.

Depression can affect thoughts, mood, and physical health and is characterized by low mood, sadness, insomnia, lack of energy, and an inability to experience pleasure [18]. Human depressive phenotypes and mouse behaviors have overlapping characteristics, so behavioral tests in mice are used to assess and quantify the extent of depressive‐like behaviors, among which sucrose preference test (SPT), female urine sniff test (FUST) and forced swim test (FST) are most widely used [19, 20, 21]. SPT aims to assess anhedonia, which is the core symptom of depression and represents the inability to experience pleasure from enjoyable activities and rewarding [22]. FUST is one of the most commonly used depressive‐like behavior assays by assessing reward‐seeking behavior [23]. Immobility, the posture to reflect the state of “behavioral despair” in which animals no longer escape, is thought to be related to depression and assessed by FST [24].

In the present study, we investigate the effect and possible mechanism of IL‐17A on depressive‐like behaviors of psoriasiform dermatitis mice and evaluate the alleviation effect of IL‐17A neutralization on its neuronal inflammatory reaction and depressive‐like behaviors to demonstrate further the pathogenic effect of IL‐17A on the psychological comorbidity of depression in psoriasis patients.

## 2 Materials and Methods

### 2.1 Mice and Treatment

**Figure 1:**
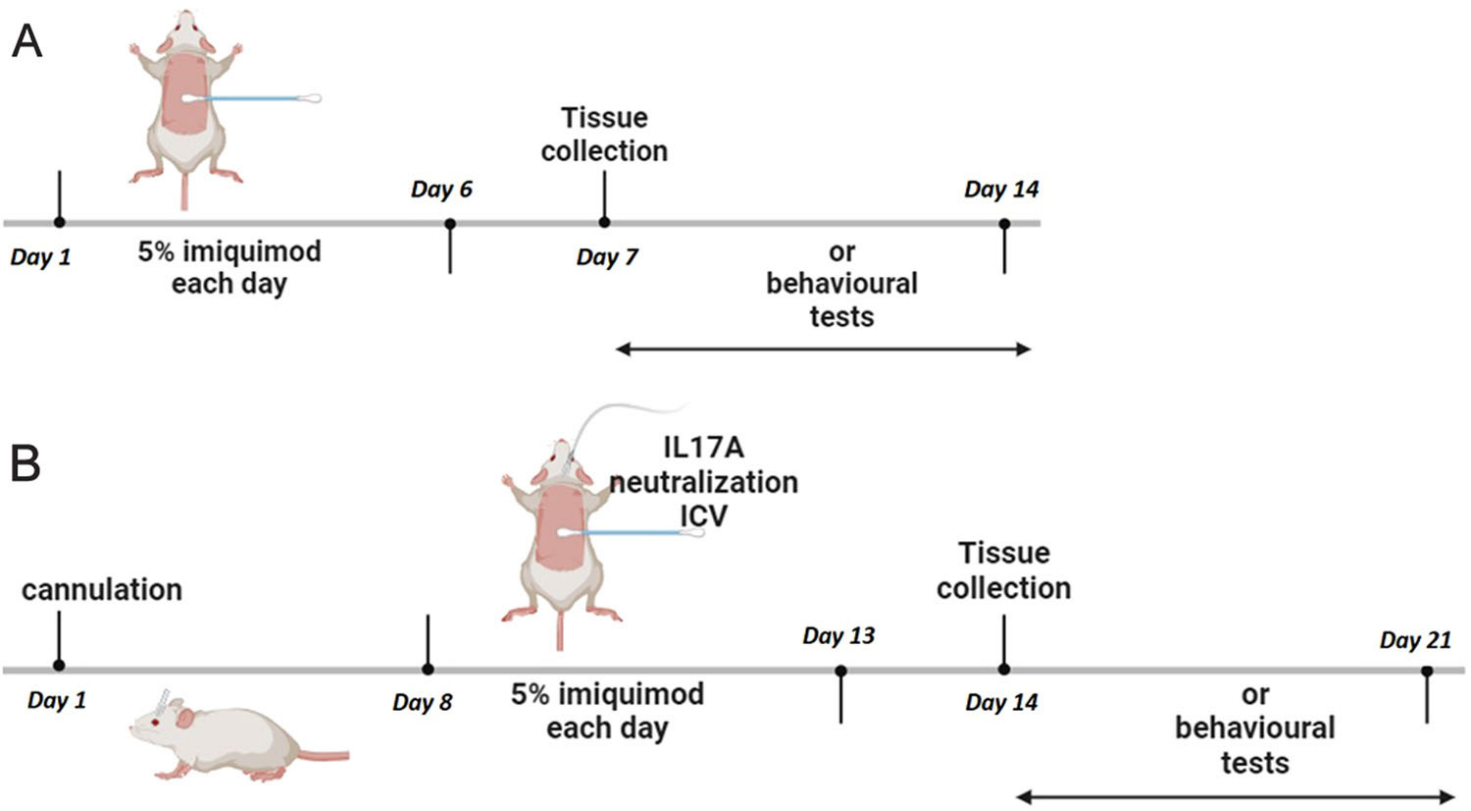
Experimental design and procedures. (A) To assess the depressive‐like symptoms of psoriasiform dermatitis mice, IMQ was topically applied on the shaved backs of experimental mice for 6 consecutive days, then on the seventh day, behavioral tests were conducted, and the skin, serum, and brain samples were collected for subsequent experiments. (B) To investigate the interventional effect of neutralization of IL‐17A on depressive‐like symptoms of psoriasiform dermatitis mice, lateral ventricular catheterization was performed before the establishment of IMQ‐induced psoriasiform dermatitis. After 7 days of recovery, experimental mice were scheduled the following model establishment, tissue collection, and behavioral assessment.

Male BALB/c mice (aged 6–8 weeks, weighed 16–20 g; purchased from Jinan Pengyue Laboratory Animal Breeding Co. Ltd.) were maintained in groups of two to three animals per cage and bred in a specific pathogen‐free environment at 22 ± 2°C with a 12‑h light/dark cycle in the animal center of Binzhou Medical University Hospital. For the development of psoriasiform dermatitis, 5% imiquimod (IMQ) cream (62.5 mg; Sichuan Mingxin Pharmaceutical Co. Ltd.) was topically applied on the shaved backs of experimental mice for 6 consecutive days, while the control mice were treated by an equivalent quantity of vaseline (Qingdao Hainuo Biological Engineering Co. Ltd.). To assess the possible effects of IL‐17A intervention on the depressive‐like symptoms of experimental mice, lateral ventricular catheterization was performed in advance for receiving an intracerebroventricular injection of anti‐IL‐17A antibody (0.02 μg/μL, 1 μL; cat. no. MAB421, R&D Systems, USA) or isotype control antibody (0.02 μg/μL, 1 μL; cat. no. A7028, Beyotime Biotechnology) for 6 consecutive days, and at the same time, IMQ or vaseline was topically applied on their shaved backs. On the seventh day, experimental mice were administered to be euthanized and then performed the following experimental procedures (Figure 1). Serum samples were obtained by heart puncture for IL‐17A level measurement, skin tissues were collected for histopathological examination, and brain tissues were acquired for cytokine level detection by RT‐qPCR and for microglia and newly generated hippocampal neurons analysis by immunofluorescent assay. All the animal procedures were approved by the Laboratory Animal Ethics Committee of Binzhou Medical University Hospital (Approval No. 20190104‐15) and carried out in accordance with the UK Animals (Scientific Procedures) Act, 1986, and ARRIVE guideline, and the EU Directive 2010/63/EU for animal experiment.

### 2.2 Stereotaxic Surgery

For intraventricular cannula implantation, mice were anesthetized with a cocktail (0.1 mL/kg, intramuscular) containing xylazine 8 mg/mL, ketamine 60 mg/mL, and acepromazine 1 mg/mL, and mounted onto a stereotaxic frame (KOPF, USA). Guide cannula was firstly inserted into the lateral ventricle (coordinates: M‐L/A‐P/D‐V, 1.1 mm/−0.2 mm/−1.6 mm). Mice were then housed individually and allowed to recover for 7 days. Text, a 33‐G stainless‐steel injector with a 5‐μL syringe was inserted into the guide cannula and extended 1 mm beyond its tip to implement intracerebroventricular injection. Drugs or vehicles were infused in a volume of 1 μL over 5 min.

### 2.3 Skin Structural Characteristics Assessment and Histopathological Examination

Skin structural characteristics and severity were assessed by the target lesion scores referring to the clinical psoriasis area and severity index (PASI) [9]. Erythema, scaling, and thickness were scored on a scale from 0 to 4. The cumulative score of the three indicators from 0 to 12 served as a measure of the severity of psoriasiform dermatitis.

Histopathological examination was performed with hematoxylin and eosin stain using standard procedures and evaluated by experienced pathologists in a double‐blinded manner. The thickness of epidermal cell layers was measured by the Image‐Pro Plus 6.0 imaging system.

### 2.4 Behavioral Tests

#### 2.4.1 Sucrose Preference Test

Before testing, mice were firstly habituated to drinking water from two bottles for 1 week. On the test day, after deprivation of water for 3 h, mice have the free choice of either drinking 1% sucrose solution or water for 2 h. The consumption of water and sucrose was measured, and the sucrose preference was calculated as the ratio of the sucrose consumption versus the total consumption of sucrose and water.

#### 2.4.2 Female Urine Sniff Test

The test was conducted as the following procedure: 3‐min exposure to the cotton tip dipped in water, 45‐min interval, and 3‐min exposure to the cotton tip impregnated with fresh urine from pubescent female mice. The duration of female urine sniffing time was scored.

#### 2.4.3 Forced Swim Test

Mice were placed in a plexiglas cylinder (25 cm height × 10 cm diameter) filled with water at a 15 cm depth (24°C ± 1°C) and their behaviors were recorded by a camera positioned directly above the cylinder for 6 min. No movement of the limb or body except those caused by respiration was defined as immobility and the duration of immobility in the last 4 min was measured.

#### 2.4.4 Locomotor Activity Test

Mice were placed in open field cages (40 × 40 × 30 cm) for 10‐min free exploration under illuminated conditions. The total traveled distance (locomotor activity) was quantified using Fusion v6.5 M software (Omnitech Electronics Inc., Columbus, OH).

### 2.5 Enzyme‐Linked Immunosorbent Assay (ELISA) for IL‐17A Serum Level Detection

Blood samples were acquired by cardiac puncture and sera were collected. IL‐17A concentration was quantified by commercialized ELISA kits according to the manufacturer's instruction (R&D Systems, Minneapolis, MN, USA).

### 2.6 Reverse Transcription‐Quantitative Polymerase Chain Reaction (RT‐qPCR)

Total RNA extraction from the hippocampus or cortex was performed with TRIzol (cat. no. 15596018; Thermo Scientific™, USA), and reverse transcription into cDNA was conducted utilizing RevertAid First Strand cDNA Synthesis Kit (cat. no. K1622; Thermo Scientific™). RT‐qPCR was performed on the StepOnePlus™ Real‐Time PCR System (Applied Biosystems, USA) with the following primers (Table 1) and AceQ qPCR SYBR Green Master Mix (cat. no. Q141‐02; Vazyme Biotech, China). The expression levels of target genes were calculated based on the following formula 2^−ΔΔCt (ΔΔCt = ΔCttreated − ΔCtcontrol)^.

**Table 1:** Primer sequences of IL‐1β, IL‐6, TNF‐α, IL‐4, IL‐10 and β‐actin.

Gene	Primer sequence
IL‐1β	Sense: 5′‐CAAGGCCACAGGTATTTTGT‐3′ Antisense: 5′‐GAAATGCCACCTTTTGACAG‐3′
IL‐6	Sense: 5′‐GGCCTTCCCTACTTCACAAG‐3′ Antisense: 5′‐ATTTCCACGATTTCCCAGAG‐3′
TNF‐α	Sense: 5′‐CAGCCTCTTCTCATTCCTGCTTGTG‐3′ Antisense: 5′‐CTGGAAGACTCCTCCCAGGTATAT‐3′
IL‐4	Sense: 5′‐GGCATTTTGAACGAGGTCAC‐3′ Antisense: 5′‐AAATATGCGAAGCACCTTGG‐3′
IL‐10	Sense: 5′‐ATTTGAATTCCCTGGGTGAGAAG‐3′ Antisense: 5′‐CACAGGGGAGAAATCGATGACA‐3′
β‐Actin	Sense: 5′‐AGCCATGTACGTAGCCATCC‐3′ Antisense: 5′‐TGTGGTGGTGAAGCTGTAGC‐3′

### 2.7 Immunofluorescent Assay

Brain samples were fixed in 4% paraformaldehyde at 4°C overnight, then dehydrated in 30% sucrose for 48 h. Coronal sections (40 μm) containing the target brain region were obtained by using the freezing microtome (CM1950; Leica, Germany). After incubated with blocking buffer for 1 h, sections were subjected to primary antibodies (IBA‐1, cat. no. ab289874, 1:800 Abcam, USA; DCX, cat. no. ab18723, 1:1000, Abcam, USA) overnight at 4°C, then incubated with secondary antibody for 4 h at room temperature. Images were captured by the Olympus FV10 confocal system. The area coverage of IBA‐1‐positive cells was quantified. For DCX quantification, the cells located within the whole dentate gyrus (DG) granule cell layer were counted.

### 2.8 Statistical Analysis

Data are presented as mean ± standard error of the mean. The normality and equal variance assumptions were tested by Shapiro–Wilk test and *F* test, respectively. For normally distributed data, two‐tailed *t* test or two‐tailed *t* tests with Welch's correction was performed to assess differences between two groups with equal variance or unequal variance, respectively. Comparison analysis among three groups was conducted by one‐way analyses of variance followed by Tukey's multiple comparison test. For non‐normally distributed data, Mann–Whitney *U* tests and Kruskal–Wallis test followed by Dunn's multiple comparison test were performed to compare differences between two groups and among three groups, respectively. IBM SPSS Statistics 25.0 and GraphPad Prism 9.5.1 were employed for statistical analysis. *p* < 0.05 was considered statistically significant.

## 3 Results

### 3.1 IMQ Treatment Induced Typical Psoriasiform Dermatitis With Increased Serum IL‐17A Levels

**Figure 2:**
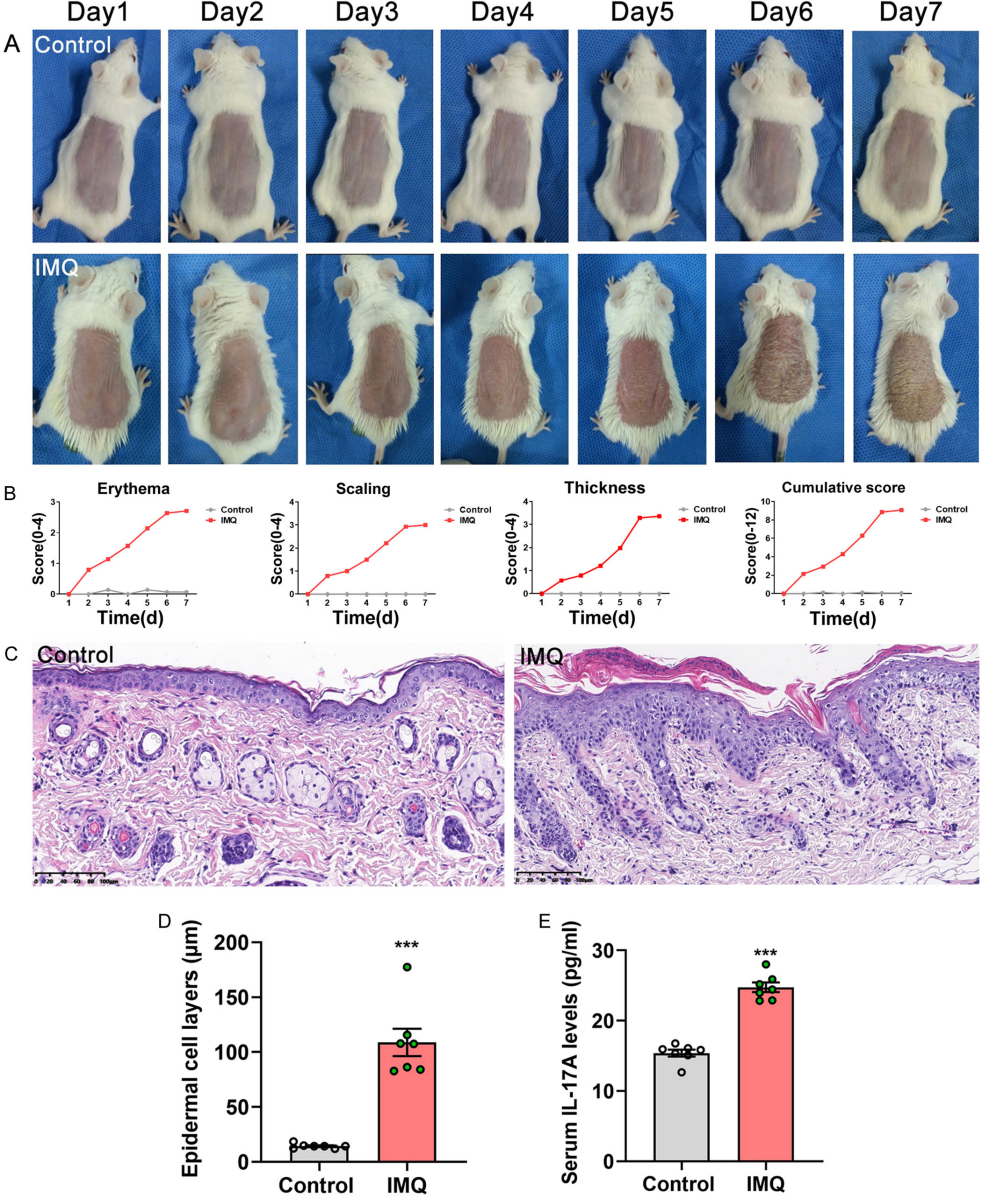
Skin structural and pathological characteristics as well as serum IL‐17A levels of experimental mice. (A) Skin structural characteristics of experimental mice. Control mice displayed no signs of psoriasiform dermatitis. Model mice presented significant erythema, scaling, and thickening characteristics. (B) Target lesions’ scores of experimental mice. Erythema, scaling, thickness, and cumulative scores of model mice were all significantly higher than control mice. (C) Skin pathological characteristics by hematoxylin and eosin staining (magnification: ×200). Control mice displayed only 1–2‐layer epidermal cells. Model mice presented hyperkeratosis, parakeratosis with Munro's micro‐abscesses, epidermal hyperplasia with trochanteral extension, dermal telangiectasia, and inflammatory cell infiltration. (D) The epidermal cell layers of model mice were remarkably thicker than control mice. (E) The serum IL‐17A levels of model mice were obviously higher than control mice. Compared with control, ***p < 0.001.

IMQ treatment developed significant signs of psoriasiform dermatitis in model mice, which was also confirmed by the scores of target lesions and histopathological examination (Figure 2A–C). The epidermal cell layers of model mice were obviously thicker than those of control (108.77 ± 33.07 µm vs. 14.28 ± 2.10 µm, *t* = 7.544, *p* < 0.001, each *n* = 7, Figure 2D). In addition, serum IL‐17A concentrations of model mice were obviously higher than those of control mice (24.74 ± 1.82 pg/mL vs. 15.38 ± 1.32 pg/mL, *t* = 11.008, *p* < 0.001, each *n* = 7, Figure 2E).

### 3.2 Psoriasiform Dermatitis Mice Presented Significant Depressive‐Like Symptoms

**Figure 3:**
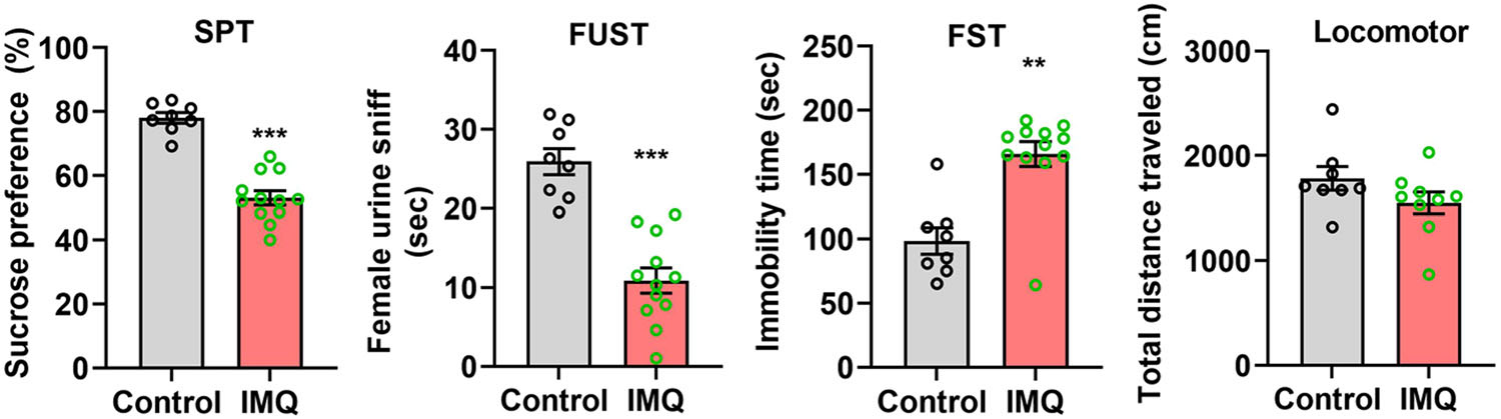
IMQ‐induced psoriasiform dermatitis mice presented prominent depressive‐like behaviors, while did not be accompanied by locomotor activity change. Sucrose preference test (SPT), Female urine sniff test (FUST), Forced swim test (FST), Locomotor activity test. Compared with control, ***p < 0.001, **p < 0.01.

Behavioral tests were conducted 24 h later after the last time administration of IMQ or vaseline. Compared with the control mice (*n* = 7), psoriasiform dermatitis mice (*n* = 12) displayed prominent depressive‐like behaviors, including decreased SPT (53.09 ± 7.539% vs.78.04 ± 4.646%, *t* = 8.322, *p* < 0.001, Figure 3), shorted FUST (10.88 ± 5.517 s vs. 25.90 ± 4.659 s, *t* = 6.330, *p* < 0.001, Figure 3) and longed FST (165.8 ± 9.744 s vs. 98.38 ± 10.36 s, *Z* = −3.086, *p* < 0.01, Figure 3). However, locomotor activity was not affected (1549 ± 105.6 cm vs. 1783 ± 318.7 cm, *t* = 1.516, *p* > 0.05, Figure 3).

### 3.3 Psoriasiform Dermatitis Mice Displayed Remarkably Increased Microglial Activation and Impaired Hippocampal Neurogenesis

**Figure 4:**
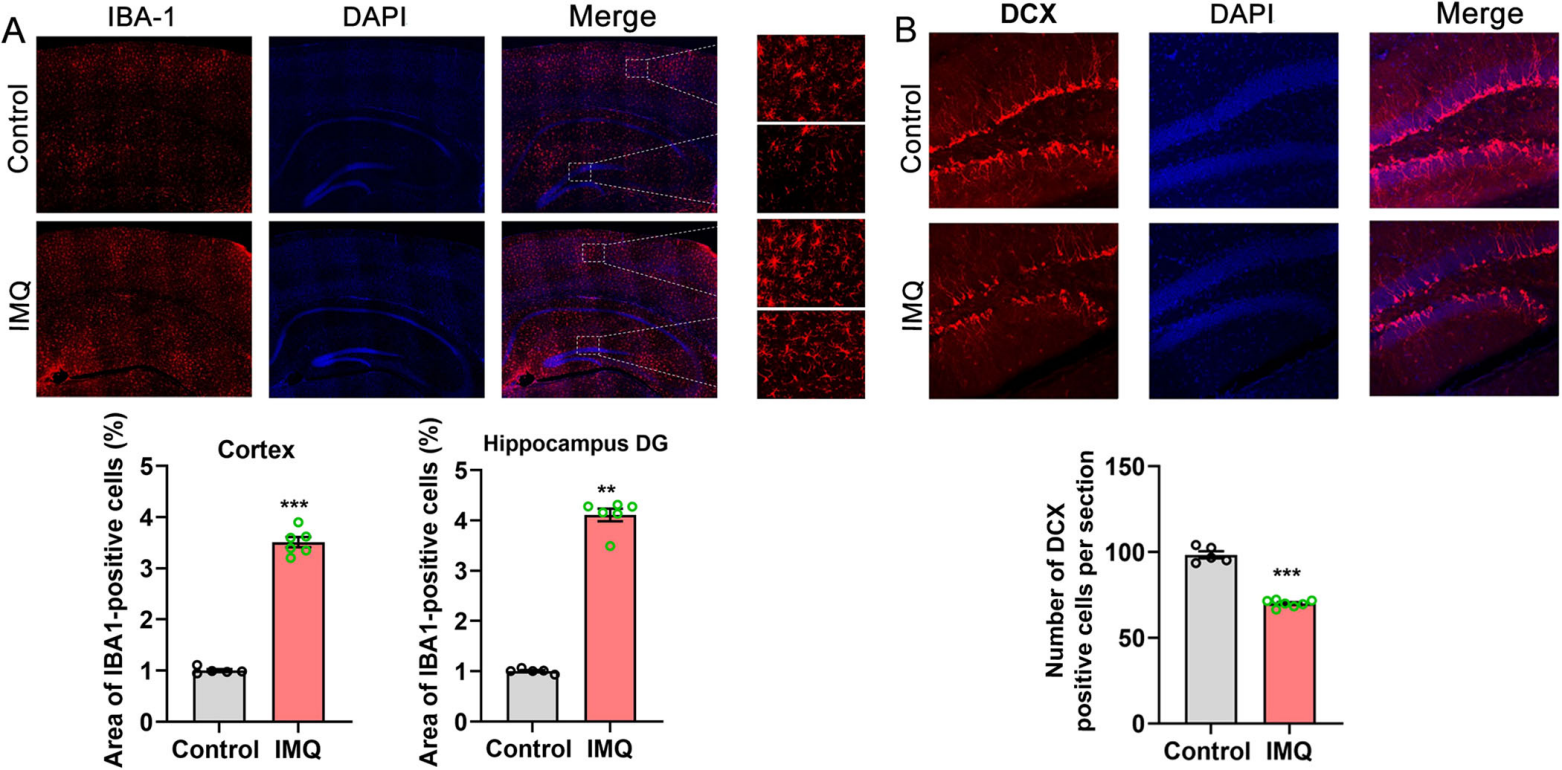
IMQ‐induced psoriasiform dermatitis mice presented significant microglia activation and hippocampal neurogenesis inhibition. (A) Immunofluorescent staining of IBA‐1‐positive cells in the cortex and hippocampal DG (compared with control, ***p < 0.001, **p < 0.01). (B) Immunofluorescent staining of DCX‐positive cells in hippocampal DG. Compared with control, ***p < 0.001.

Ionized calcium‐binding adapter molecule 1 (IBA‐1) is specifically expressed in microglia and plays an important role in regulating the function of microglia, especially in the activated microglia [25]. Immunofluorescent results showed that the area of IBA‐1‐positive cells in the cortex and hippocampal DG of psoriasiform dermatitis mice (*n* = 6) were notably increased compared with control mice (*n* = 5) (cortex, 3.509 ± 0.1007% vs. 1.003 ± 0.02808%, *t* = 23.990, *p* < 0.001; hippocampal DG, 4.106 ± 0.1269% vs. 1.005 ± 0.02132%, *Z* = −2.739, *p* < 0.001, Figure 4A). The reduction of hippocampal neurogenesis is implicated in depression development. Doublecortin (DCX) is a microtubule‐associated protein and critical for normal neuronal migration during development, which has been widely used as a reliable marker to study post‐mitotic, immature neurons in the adult mammalian brain and considered as a proxy for the level of adult neurogenesis [26]. DCX staining was conducted to evaluate hippocampal neurogenesis in the resign of DG. The number of DCX‐positive cells was significantly decreased in psoriasiform dermatitis mice (*n* = 7) compared with control mice (*n* = 5) (69.88 ± 0.778 vs. 98.33 ± 2.062, *t* = 14.620, *p* < 0.001, Figure 4B).

### 3.4 Pro‐Inflammatory Cytokines Production Is Notably Upregulated in the Cortex and Hippocampus of Psoriasiform Dermatitis Mice

**Figure 5:**
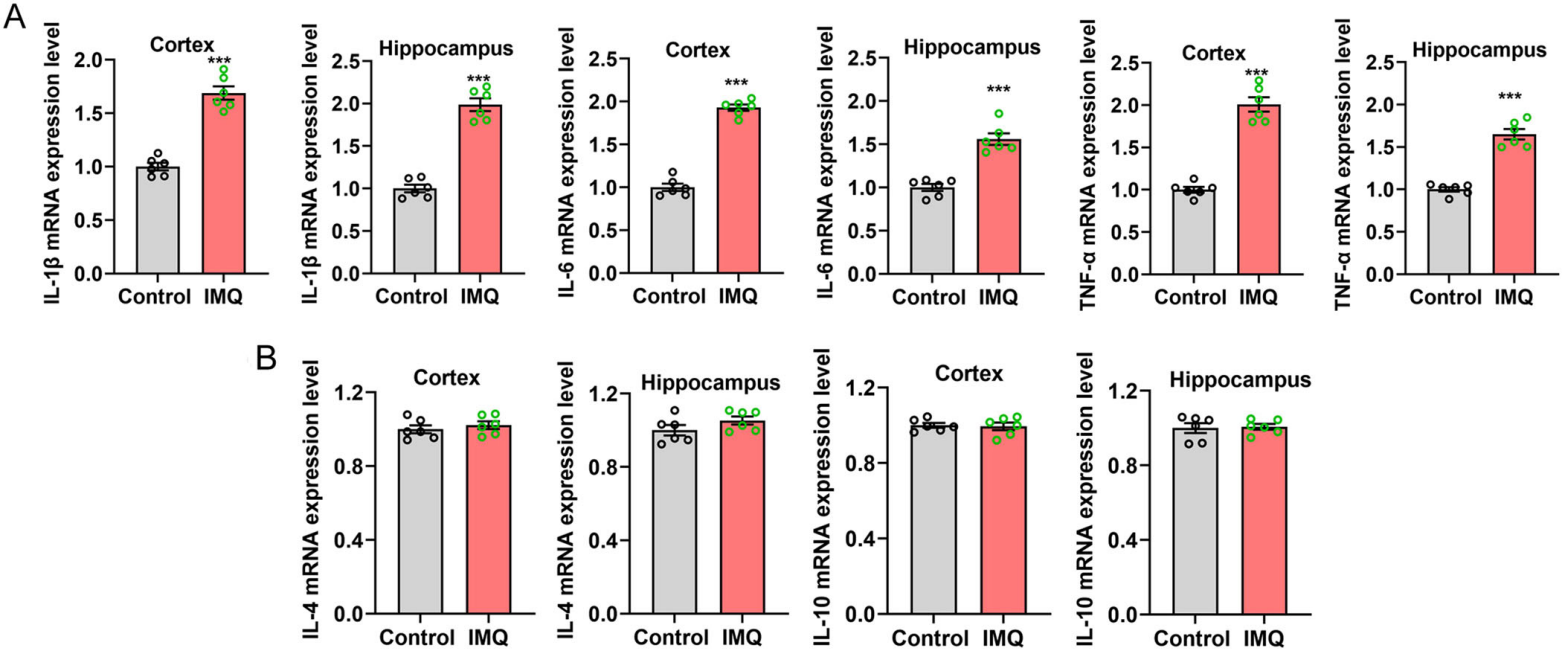
Pro‐inflammatory cytokines expression levels were significantly increased in the hippocampus and cortex of psoriasiform dermatitis mice. (A) Pro‐inflammation cytokines IL‐1β, IL‐6, and TNF‐α expression levels. (B) Anti‐inflammatory factors IL‐4 and IL‐10 expression levels. Compared with control, ***p < 0.001.

The mRNA expression levels of pro‐inflammatory factors IL‐1β, IL‐6, and TNF‐α in the cortex and hippocampus were obviously elevated in psoriasiform dermatitis mice compared with control mice (each *n* = 6, IL‐1β, cortex 1.688 ± 0.063 vs. 1.000 ± 0.035, *t* = 9.513, *p* < 0.001, hippocampus 1.986 ± 0.074 vs. 1.000 ± 0.045, *t* = 11.370, *p* < 0.001; IL‐6, cortex 1.929 ± 0.037 vs. 1.000 ± 0.043, *t* = 16.520, *p* < 0.001, hippocampus 1.560 ± 0.067 vs. 1.000 ± 0.041, *t* = 7.167, *p* < 0.001; TNF‐α, cortex 2.008 ± 0.085 vs. 1.000 ± 0.034, *t* = 11.030, *p* < 0.001, hippocampus 1.650 ± 0.061 vs. 1.000 ± 0.026, *t* = 9.730, *p* < 0.001, Figure 5A). However, there was no significant difference in the expression of anti‐inflammatory factors between psoriasiform dermatitis mice and control mice (IL‐10, cortex 0.995 ± 0.020 vs. 1.000 ± 0.012, *t* = 0.221, *p* > 0.05, hippocampus 1.007 ± 0.015 vs. 1.000 ± 0.027, *t* = 0.219, *p* > 0.05; IL‐4, cortex 1.023 ± 0.021 vs. 1.000 ± 0.022, *t* = 0.740, *p* > 0.05, hippocampus 1.053 ± 0.022 vs. 1.000 ± 0.028, *t* = 1.488, *p* > 0.05, Figure 5B).

### 3.5 Neutralization of IL‐17A in Lateral Ventricular Exerted No Measurable Effects on the Severity Degree of Psoriasiform Dermatitis and Serum IL‐17A Levels

**Figure 6:**
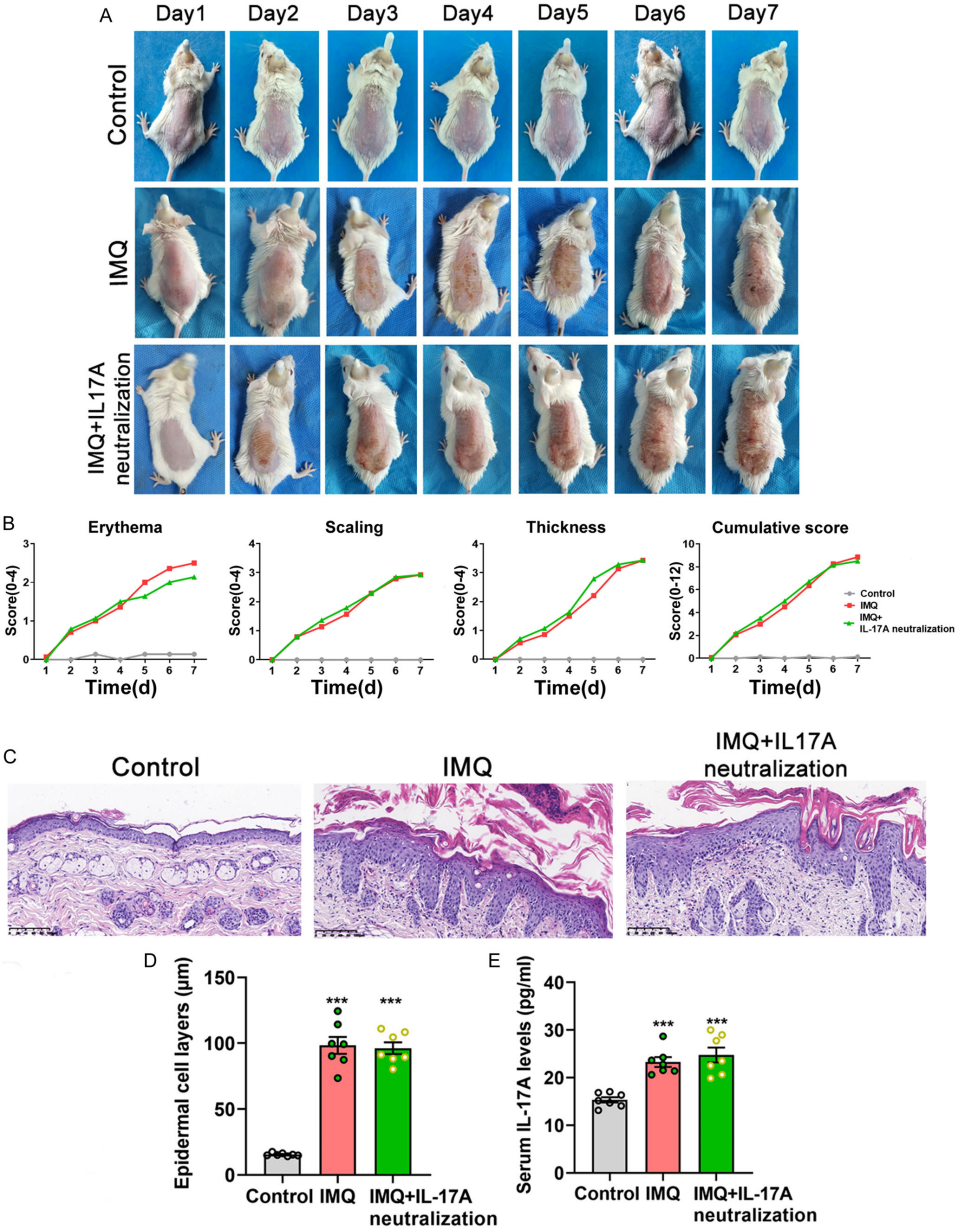
Neutralization of IL‐17A in lateral ventricular exerted no influence on model mice's skin structural and histopathological characteristics and IL‐17A serum levels. (A) Skin structural characteristics. (B) Erythema, scaling, thickness, and cumulative scores. (C) Histopathological characteristics. (D) The epidermal cell layers. (E) IL‐17A serum levels. Compared with control, ***p < 0.001.

The skin structural and histopathological characteristics and target lesions scores presented similarly in only the IMQ group and IMQ plus neutralization of the IL‐17A group (Figure 6A–C), and there was no significant difference in the cumulative target lesion scores (8.90 ± 0.90 vs. 8.50 ± 1.08, *p* = 0.515), epidermal cell layers (98.41 ± 17.00 µm vs. 96.20 ± 11.72 µm, *p* = 0.782) and serum IL‐17A levels (23.28 ± 2.71 pg/mL vs. 24.77 ± 4.11 pg/mL, *p* = 0.440) between the above two groups (each *n* = 7, Figure 6B,D,E).

### 3.6 Neutralization of IL‐17A in Lateral Ventricular Significantly Alleviated the Depressive‐Like Behaviors of Psoriasiform Dermatitis Mice

**Figure 7:**
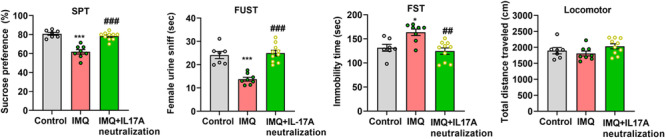
Neutralization of IL‐17A in lateral ventricular significantly alleviated the depressive‐like behaviors of psoriasiform dermatitis mice, without affecting their locomotor activity. (A) SPT, (B) FUST, (C) FST, (D) Locomotor activity test. Compared with control, ***p < 0.001, *p < 0.05; compared with IMQ, ^*###*^ *p* < 0.001, ^*##*^ *p* < 0.001.

The differences were significant in the behavioral tests of SPT, FUST, and FST among the control group, IMQ group, and IMQ plus neutralization of IL‐17A group (*n* = 7, 8, 10 respectively; *F* = 33.820, *F* = 26.290 and *H* = 9.832, *p* < 0.001 or *p* < 0.01, Figure 7). Multiple comparisons showed that neutralization of IL‐17A treatment could significantly alleviate depression levels (*p* < 0.01 or 0.001), and the performances of IMQ plus neutralization of IL‐17A group were similar to the control group (all *p* > 0.05). While, in the locomotor activity test, there were no differences among the three groups (*F* = 2.011, *p* > 0.05).

### 3.7 Neutralization of IL‐17A Inhibited Microglia Activation, but Promoted Hippocampal Neurogenesis

**Figure 8:**
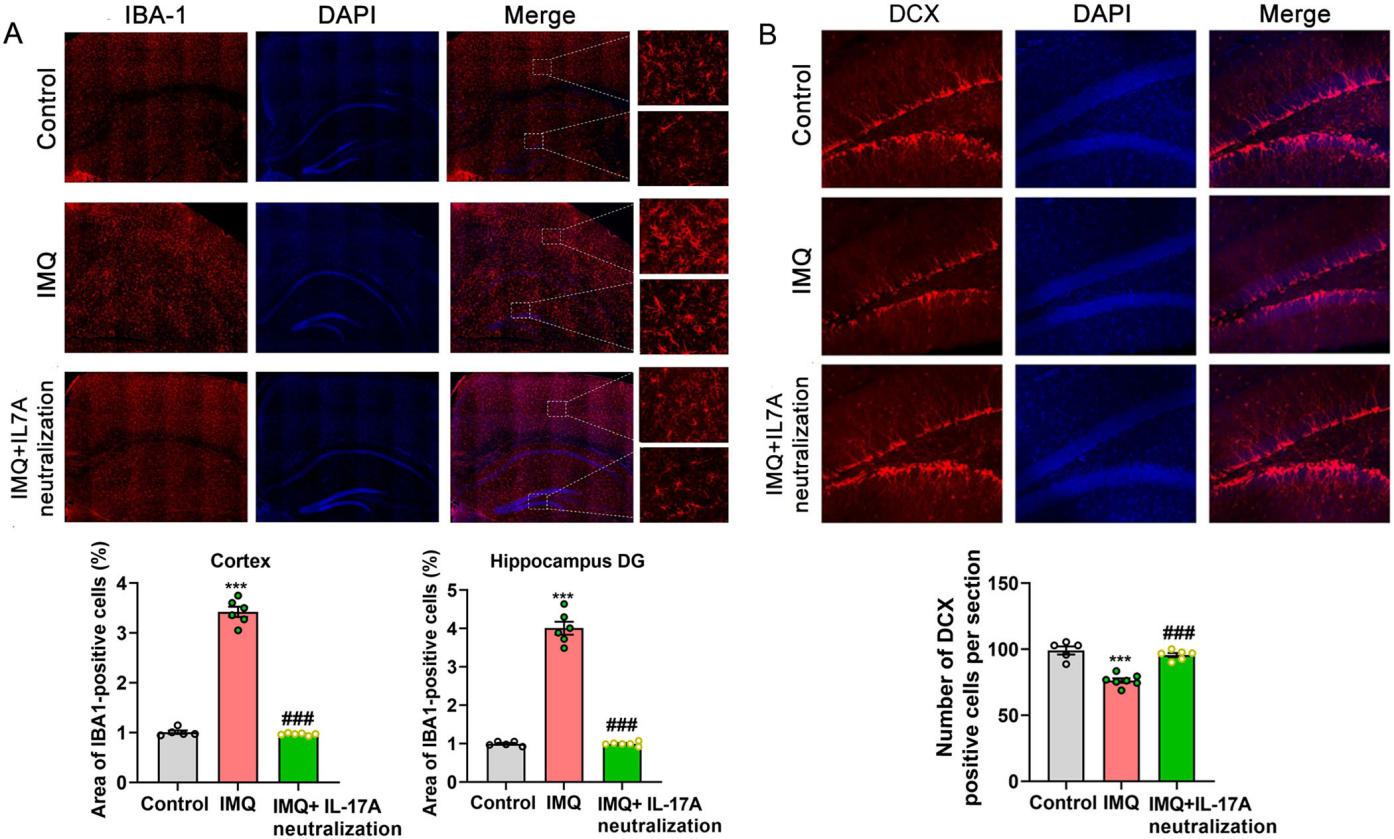
Neutralization of IL‐17A inhibited microglia activation, but promoted hippocampal neurogenesis. (A) IBA‐1‐positive cells in the cortex and hippocampal DG. (B) DCX‐positive cells in the hippocampal DG. Compared with control, ***p < 0.001; compared with IMQ, ^###^ *p* < 0.001.

IBA1‐positive cells in the cortex and hippocampal DG were significantly different among the control group, IMQ group, and IMQ plus neutralization of IL‐17A group (*n* = 5, 6, 6, respectively; *F* = 477.0 and *F* = 282.2, *p* < 0.001, Figure 8A). Between‐group comparison revealed that treatment of IL‐17A neutralization in lateral ventricular could obviously reduce the number of IBA1‐positive cells and inhibit microglia activation (*p* < 0.001), which were close to the control group (*p* > 0.05, Figure 8A). The newly generated neurons in the hippocampus DG stained by DCX were significantly different in the three experimental groups (*n* = 5, 7, 6, respectively; *F* = 37.90, *p* < 0.001), and the obvious inhibition of neurogenesis in IMQ‐treated mice was reversed by neutralization of IL‐17A (*p* < 0.001), which appeared similar to the control group (*p* > 0.05, Figure 8B).

### 3.8 Neutralization of IL‐17A Suppressed Pro‐Inflammatory Factors Production

**Figure 9:**
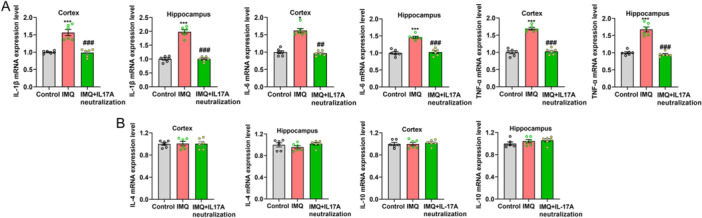
Neutralization of IL‐17A in lateral ventricular suppressed pro‐inflammatory factors production. (A) Pro‐inflammatory cytokines IL‐1β, IL‐6, and TNF‐α mRNA expression levels. (B) Anti‐inflammatory factors IL‐4 and IL‐10 mRNA expression levels. Compared with control, ***p < 0.001, *p < 0.05; Compared with IMQ, ^*###*^ *p* < 0.001, ^*##*^ *p* < 0.001.

The differences of pro‐inflammation cytokines IL‐1β, IL‐6, and TNF‐α mRNA expression in the fields of cortex and hippocampus were all significant among the control group, IMQ group and IMQ plus neutralization of IL‐17A group (*n* = 5, 6, 6, respectively; cortex, *F* = 31.910, *H* = 11.556, and *F* = 102.600; hippocampus, *F* = 107.800, 46.680, and 71.520, *p* < 0.001 or 0.01 respectively, Figure 9A). Besides the significant differences between control mice and IMQ‐treated mice, the abnormal increased mRNA expression levels of pro‐inflammation cytokines in IMQ‐treated mice were certainly descended by neutralization of IL‐17A treatment (*p* < 0.01 or 0.001), and the expression differences were slight between the control group and IMQ plus IL‐17A neutralization group (*p* > 0.05). In addition, the mRNA expression levels of anti‐inflammatory factors IL‐4 and IL‐10 among the three experimental groups showed no statistical difference (all *p* > 0.05, Figure 9B).

## 4 Discussion

Psoriasis is an immune‐mediated systemic inflammatory disorder, which is highly concerned not only owing to the visible lesions but also accompanying comorbidities, such as psoriatic arthritis, kidney disease, gastrointestinal diseases, cardiovascular disease, metabolic syndrome, and psychological disorders [2]. Compared to the general population, patients with psoriasis have higher rates of depression, anxiety, and suicidality, moreover, their prevalence in psoriasis patients is even higher than in patients with other dermatological conditions [6, 7]. Although the association between psychological comorbidities and psoriasis is well known, the links between them have not been fully identified. To address the above possible pathogenic links, an IMQ‐induced mouse model of psoriasis was established, which mimics many features of human psoriasis. Psoriasis is considered to be mainly dominated by Th17 cells and its effective cytokine IL‐17A [8]. In clinical practice, monoclonal antibodies blocking either IL‐17A or its receptor have been confirmed to be the first‐line treatment for moderate‐to‐severe plaque psoriasis (the clinical PASI score> 10) [27]. IMQ‐induced mouse psoriasis model has been demonstrated to be mediated via the IL‐23/IL‐17 axis [9]. In the present study, IMQ‐induced psoriasiform dermatitis mice presented typical psoriatic pathological characters and obviously elevated IL‐17A serum levels, which further confirmed the pathogenic effects of IL‐17A in the pathogenesis of psoriasis. In addition, the model mice were confirmed significant depressive‐like behaviors by SPT, FUST, and FST assessment. An ample number of studies have shown the interaction between the central nervous system and the immune system. Some inflammatory cytokines can induce a cascade of events in the brain by crossing the blood‐brain barrier and play a key role in the pathogenesis of depression [28]. Although psoriasis and depression retain their own unique set of cytokines in their respective pathogenesis, IL‐17 may participate in both of the pathogenesis [29]. While, it is not clear if psoriasis‐induced systemic IL‐17A increase can mediate the neuronal inflammation and result in depressive‐like symptoms. The study of nitroglycerin‐induced chronic migraine and myocardial infarction has shown that IL‐17 can cross the blood‐brain barrier to trigger neuroinflammation, which might be the key factor for their corresponding depression performance [30, 31]. Microglia are the first line of the innate immune system in the central nervous system, and depression is considered a microglia‐associated disorder (microgliopathy) [32, 33]. The receptor for IL‐17A is expressed in microglia [34, 35] and IL‐17A has been shown to impair the brain‐blood barrier integrity and to activate microglia [36]. Microglial activation can be divided into the classic M1 polarization or alternate activation of M2 under optimal conditions. M1 microglia secretes pro‐inflammation mediators IL‐1β, IL‐6, and TNF‐α, and M2 microglia is inclined to express anti‐inflammation cytokines IL‐4 and IL‐10, which is driven by the coordinated regulation of multiple anti‐inflammatory mediators [37, 38]. IL‐17A has been demonstrated to be involved in inducing the production of IL‐1β, IL‐6, and TNF‐α [30]. Activation of microglia in different brain regions, including the prefrontal cortex (PFC), hippocampus, anterior cingulate cortex (ACC), and amygdala, are involved in the development of depression. In the present study, microglia activation in IMQ‐induced psoriasiform dermatitis mice was verified in the fields of hippocampus DG and cortex and substantial production of pro‐inflammation cytokines IL‐1β, IL‐6, and TNF‐α, while there was no significant difference in IL‐4 and IL‐10 expression levels, which further indicates that IL‐17A‐induced systemic inflammation can contribute to the development of a depressive‐like state, and in the pathological condition of psoriasis, systemic IL‐17A can trigger depression by mediating microglia M1 polarization and neuroinflammation. On the other hand, microglia can also produce IL‐17A in pathological conditions [39, 40]. In the neuroinflammatory response to stress, surveying CD4^+^ T cells may differentiate into Th17 cells in situ especially when receiving signals from pro‐inflammatory cytokines such as IL‐1β, IL‐6, and TNF [15]. Since these above cytokines are known to promote Th17 cell differentiation, we may speculate that in the pathological condition of psoriasis, the release of pro‐inflammation mediators of IL‐6, IL‐1β, and TNF‐α may in turn impose on microglia and local immune cells to produce IL‐17A, then further aggravate the neuroinflammation and depression development.

Adult hippocampal neurogenesis plays a key role in modulating memory and emotion processing [41]. Microglia activation has been reported to be a pivotal mechanism in neurogenesis inhibition in the presence of inflammation and stress, which can strongly suppress adult hippocampal neurogenesis by decreasing neuroprogenitor proliferation, newborn cell survival, and impairing newborn neuron maturation [32, 42, 43]. The adult mammalian hippocampus contains quiescent neural stem cells in the DG that upon activation generate new neurons [41]. In this study, we also demonstrated the distinct decrease of newly generated hippocampal neurons in the DG, which further confirms that the depressive‐like symptoms in IMQ‐induced psoriasiform dermatitis mice are mediated by neuroimmune inflammation solidly.

To further clarify the critical role of IL‐17A in triggering depression in psoriatic disease, intracerebroventricular injection of anti‐IL‐17A antibody was implemented during the period of IMQ application for the establishment of psoriasiform dermatitis, which is a technologically matured administration way in animal experiments of psychiatric and psychological disorders, and also commonly used in the screen research of antidepressant drugs and mechanism exploration [44, 45, 46, 47]. Compared to the system administration method, such as intravenous injection, intracerebroventricular injection may reduce the potentially indirect effects on depressive‐like behaviors of experiment mice to the minimum caused by systemic administration on other tissues and organs. There was no significant difference in psoriasiform dermatitis and serum IL‐17A levels between the two treatment methods of IMQ and IMQ plus anti‐IL‐17A antibody intracerebroventricular injection. The most probable reason for the lack of differences in the severity of skin lesions, histopathological characters, and IL‐17A serum levels between the two groups may be due to the limited lateral ventricular capacity and very low injection dosage of anti‐IL‐17A antibody (0.02 μg/μL, 1 μL), which was restricted to neutralize the increased IL‐17A in brain tissue, but not enough to affect peripheral circulation and improve skin lesions. Encouragingly, the neutralization of IL‐17A significantly alleviated the depressive‐like behaviors of experimental mice. At the same time, the activation of microglia, as well as expression levels of IL‐1β, IL‐6, and TNF‐α in the field of hippocampus and cortex, were distinctly decreased, and newly generated hippocampal neurons multiplied to a great degree, which indicates the mitigation of neuroinflammation. In the study of acute cerebral ischemic injury, IL‐17A knockout or anti‐IL‐17A monoclonal antibody treatment can markedly decrease the microglial activation and induced a shift from M1 to M2 phenotypes with the decreased expression of TNF‐α, but increased IL‐10 [48]. We did not observe a significant change in the expression of anti‐inflammatory cytokines IL‐4 and IL‐10, especially when anti‐IL‐17A antibody intracerebroventricular injection was conducted, which may be due to different cytokines regulatory modes and pathways in specific disease pathological environments and needs more in‐depth research on the underlying mechanisms. Consequently, the immune‐inflammatory mechanisms are involved in both psoriasis and depression, and IL‐17A may exert a central role in triggering depression symptoms in the pathological condition. Targeted intervention of psoriasis by neutralization of IL‐17A may provide a candidate therapeutic measure for its depression comorbidity.

## 5 Conclusions

**Figure 10:**
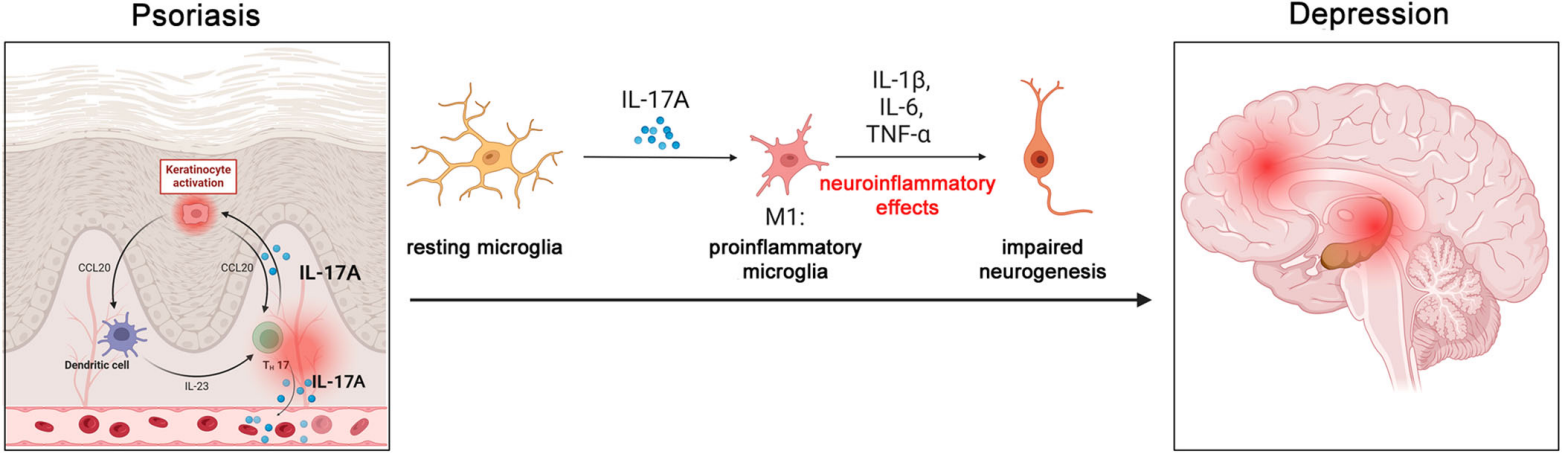
The pattern diagram of IL‐17A mediating depressive‐like symptoms by inducing microglia activation in psoriasiform dermatitis mice. In the pathological conditions, abnormal increased IL‐17A in skin lesions can enter the peripheral circulation and cross the blood‐brain barrier to mediate microglia activation and trigger neuroinflammation, then inhibit hippocampal neurogenesis and result in depressive‐like behaviors.

Our present study provides evidences that systemic IL‐17A can provoke microglia activation and neuroinflammatory effects, inhibit hippocampal neurogenesis, and trigger depression symptoms in pathological conditions of psoriasis. As an important pathogenic factor, IL‐17A contributes to its critical role in mediating systemic inflammation and comorbidities development. Although further investigations are needed to fully clarify its exact immune‐inflammation mechanism, we may believe that blocking IL‐17A may be an exactly effective approach for the treatment of psoriasis and its psychological comorbidities (Figure 10).

## Author Contributions

**Lei Ma:** conceptualization, data curation, formal analysis, supervision, funding acquisition, resources, project administration, writing‐original draft, writing‐review and editing. **Yue Dou:** formal analysis, investigation, methodology. Jingjing You: formal analysis, investigation, methodology. **Jing Wang:** investigation, methodology. **Xinxin Li:** Investigation, methodology. **Yawen Lin:** investigation, methodology. **Bin Liu:** conceptualization, project administration, formal analysis, data curation, investigation, methodology, writing‐original draft, validation.

## Ethics Statement

All animal‐related protocols were approved by the Laboratory Animal Ethics Committee of the Binzhou Medical University Hospital (Approval Number: 20190104‐15).

## Conflicts of Interest

The authors declare no conflicts of interest.

## Data Availability

All data included in this study are available upon request by contact with the corresponding author.
